# Lung cancer and Covid-19: lessons learnt from the pandemic and where do we go from here?

**DOI:** 10.1038/s41533-022-00283-x

**Published:** 2022-05-30

**Authors:** Susanne Sarah Maxwell, David Weller

**Affiliations:** Centre for Population Health Sciences, University of Edinburgh, Edinburgh, UK

**Keywords:** Health policy, Respiratory tract diseases, Public health

## Abstract

The Covid-19 pandemic has significantly disrupted all aspects of healthcare, and while the worst may be over, its broader impact on health services, such as cancer diagnosis and treatment, is likely to be profound. We examine, in this paper, how our response to Covid-19 impacted on the recognition, referral, and diagnosis of individuals with lung cancer in primary care. The overlapping nature of symptoms of Covid-19 and lung cancer posed a particular challenge, and lung cancer referrals have been slow to return to pre-pandemic levels. Strategies need to be implemented to ensure the impact of future variants does not derail the precarious recovery we are now witnessing in many countries—it is vital that the gains we have made in earlier diagnosis are not lost. The pandemic has underlined the importance of improving early diagnosis through public awareness raising of symptoms, rapid diagnostic facilities, reduced primary care diagnostic intervals and, potentially, the introduction of screening in high-risk groups.

## Introduction

Lung cancer is the leading cause of cancer death worldwide. In the UK lung cancer accounts for 21% of all cancer deaths^1^. Between 2013 and 2017, net 1- and 5-year survival following diagnosis was 40.6 and 16.2, respectively, within England^2^. While lung cancer survival rates have improved in recent decades within the UK^3^, they continue to lag behind our European neighbours^4^^,^^5^ and other developed nations^6^. Stage at diagnosis influences patient survival, with advanced disease having fewer treatment options and significantly worse survival outcomes. However, a large proportion of patients (46.1% in England) are diagnosed with stage four disease with a corresponding 5-year survival of only 2.9%.

Later stage of diagnosis has been attributed to some of the difference in survival outcomes between the UK and other developed nations but the UK has also demonstrated lower stage-specific survival^7^. Measures have been implemented to improve earlier lung cancer detection and earlier initiation of treatment. These include public awareness campaigns such as ‘Be Clear on Cancer’ in England or ‘Detect Cancer Early in Scotland’ which aims to increase public awareness of symptoms of cancer to encourage earlier presentation to primary care. Other improvements include streamlining the lung cancer pathway from primary care through to multi-disciplinary team (MDT) review and early initiation of treatment such as the ‘national optimal lung cancer pathway’^8^. Screening high-risk individuals with low-dose CT imaging is another proposed method to increase detection at earlier stage disease. There are multiple trial sites currently investigating how to implement this within the UK. Alongside these efforts to detect and treat early lung cancer, treatments of late-stage disease have improved substantially with the advent of immunotherapies—leading to survival improvements^9^.

On the 11th March 2020, the World Health Organisation (WHO) declared Covid-19 a global pandemic. This led to national lockdowns across the world and in the UK on the 23rd of March the Prime Minister issued a national message to ‘stay at home’. The knock-on consequences of these measures were felt in all walks of life. The remobilisation of the healthcare service to emergency care only in an attempt to reduce the spread of the virus and allow the NHS to cope with the influx of Covid-19 patients particularly impacted on cancer care. A WHO survey early in the pandemic found that in many countries health services for non-communicable diseases were partially or completely disrupted^10^. This included 42% of countries recognising cancer treatment being impacted. Ninety-four percent of countries described staff being partially or fully redeployed away from non-communicable diseases towards Covid-19.

While the health service is now beginning to return to normality, or the ‘new normal’, the knock-on consequences to cancer care and patient outcomes from lung cancer will likely be felt for years to come. The pandemic has had a profound impact on all aspects of the lung cancer pathway including patient presentation, diagnosis, treatment, and follow-up. Patients’ visits to treatment facilities have been curtailed, treatments have, in some cases, been withheld and there is also evidence of a negative impact on quality of life^11–13^.

## Impact of Covid-19 on potential lung cancer presentations to primary care

During the pandemic, GPs across the country and internationally have needed to adapt to new ways of working^14,15^. Busy waiting rooms where the virus could potentially spread were to be avoided during the initial phase and face to face consultations were to be limited. In the UK, GPs were advised to implement a ‘total triage’ model using telephone and online consultation tools and only provide face to face consultations that were urgently required^16^. However, ~70% of all new cancer diagnoses present first via primary care^17,18^ and in the absence of screening the majority of new lung cancer diagnoses present via this route (50–66% across the countries within the UK)^19^. Therefore, changes in access to primary care could impact on early lung cancer recognition and referral.

The first wave of the pandemic saw a reduction in the number of consultations carried out in primary care as fewer patients presented to their GP^20–22^ with a 24.1% reduction in consultations for potential cancer symptoms between 2019 and 2020 in England^23^. This was most marked during the initial lockdown period. This is unsurprising and reflects the message directed at individuals to ‘stay at home, protect the NHS and save lives’. Patients with potential cancer symptoms described ‘fear of wasting the healthcare professionals time’ and ‘worry about putting extra strain on the NHS’ as barriers to presenting during the pandemic, alongside concerns about catching and transmitting Covid-19^24^. Some patients were not even aware GP practices were open^25^.

Presentations of cough to primary care showed one of the largest sustained reductions throughout 2020^23^. This may reflect the overlapping nature of the symptom of cough with Covid-19, whereby individuals with a new cough and breathlessness were advised to isolate and get tested. Perceiving Covid-19 as the cause of symptoms was indeed found to be associated with a reduced odds of help-seeking behaviour in patients with potential cancer symptoms^24^. It is important to note, however, the implementation of Covid-19 community hubs resulted in patients with cough and other respiratory symptoms often being assessed outside of primary care so potentially under-representing this group in primary care records. Nevertheless, many patients avoided using healthcare services despite harbouring symptoms of potential concern^24^.

In line with government guidance, there was also a significant shift in type of consultations during the pandemic, with a significantly higher proportion of consultations being carried out via telephone^26,27^. While there were clear benefits to more widely used telephone triaging and remote consulting, particularly when vulnerable individuals were unvaccinated, concerns were expressed by GPs on the impact of this on early cancer detection, in particular missing subtle signs and symptoms^20^. There was a recognition of missing that ‘gut feeling’ that practitioners experience when they see a patient in person, which can be an important trigger for further investigations by GPs. A GP’s ‘gut feeling’ is increasingly being recognised as an important predictor of cancer^28,29^ and it is now incorporated into referral criteria for early cancer diagnostic centres^30,31^. Further research is needed on how remote consulting impacts on this aspect of the consultation.

Another concern about the significant shift to remote consulting was the impact on more vulnerable groups and their ability to access healthcare, potentially widening existing health inequalities. Deep End GPs were concerned about accessibility for their patients during the pandemic^32^. Socioeconomic deprivation is associated with higher incidence and poorer survival outcomes from lung cancer^33–35^ and so any increased barriers to accessing healthcare could exacerbate this inequality further.

## Impact of Covid-19 on referrals from primary care

Chest X-ray organised in primary care is the first line investigation for suspected lung cancer across the UK^36,37^. Alongside a reduction in consultations during the first wave of the Covid-19 pandemic there was also a reduction in diagnostic tests performed within primary care. A 93% reduction in chest X-rays performed was witnessed in one English hospital from 814 chest X-ray requests from primary care in April 2019 to 58 in April 2020. This effect was sustained in the following months with 957 and 655 requests in May and June 2019 compared with 78 and 217 requests in the same months in 2020^38^. This is in keeping with the reduction in diagnostic tests carried out in England between March 2020 and March 2021, where around 4.6 million fewer tests were carried out^39^.

Between March 2020 and March 2021, there were 380,000 fewer urgent suspected cancer referrals across the UK of which lung cancer accounted for 23,000^40^. April saw the sharpest reduction in urgent cancer referrals in England with an ~60% reduction in all referrals compared to April 2019^41^. After this initial drop, referral rates have gradually improved with referrals for many cancers (including breast, skin and colorectal) returning to expected levels by September 2020^42^. However, lung cancer referrals have consistently maintained below expected numbers. Between March 2020 and August 2021, 25,900 fewer patients have been referred for suspected lung cancer under the 2ww pathway in England^40^. This is a reduction of 26% over this period. While further research is needed to tease out the exact reasons behind this sustained reduction in lung cancer referrals the cause is likely multifactorial and may include the reduced presentation of symptoms such as cough to primary care, the overlapping nature of symptoms of lung cancer and Covid-19 and the presence of Covid community hubs where patients with suspicious symptoms were referred directly to secondary care.

## Impact of Covid-19 on lung cancer diagnosis and staging

The impact of Covid-19 on cancer diagnosis has been felt and documented across different countries. In Denmark, there was a 33% reduction in incident cancers at the height of the pandemic during the period between March and May 2020^43^—equivalent to ~2800 undetected cancers. They found a 24% reduction in lung cancer incidence during this time frame with the largest reduction in May 2020 of 39%. The authors estimated this was the equivalent of 238 undetected lung cancer cases. This reduction in new cancer diagnosis was similarly demonstrated in the Netherlands and Poland^44,45^. In Poland between March and May, there was a reduction of 700 respiratory and thoracic neoplasms detected compared to the same time period in 2019.

A similar impact was felt in the UK. In Scotland there was a 6.2% decrease in breast, colorectal and lung cancers diagnosed in 2019/2020 compared with 2018/2019. Between the months of April and December 2020 there were 9% fewer than expected patients diagnosed with lung cancer. This reduction was most marked during the lockdown period of March to May in keeping with other countries descriptions and gradually recovered throughout the rest of the year. By the fourth quarter of the year the numbers were at pre-pandemic expected levels^46^.

The impact of the pandemic on lung cancer stage at presentation is muted, to a degree, by the drop in overall numbers of diagnoses—but preliminary data suggest a stage-shift towards later presentation^47,48^. In Scotland, the diagnosis of all stages of disease reduced during the initial lockdown period. However, more advanced stage 3 and 4 disease returned to normal levels during the remainder of the year whereas stage 1 and 2 diagnosis increased in the later summer months when the restrictions eased and then reduced again in the winter months when Covid-19 cases were increasing and local lockdowns were imposed^46^.

There are differing possible explanations for this. Individuals with less severe symptoms may have been reluctant to attend healthcare settings when circulating virus in the community was higher, such as during the early pandemic months and later winter months. In addition, lung cancer can be picked up incidentally when undergoing a chest X-ray for other symptoms and so if patients were presenting less frequently due to fear of catching Covid-19 then incidental cancers at early stages could more likely to be missed.

## Strategies going forward

At the time of writing life has still not completely returned to normal, and new variants, such as omicron, will likely continue to appear. Across the world, different measures continue to be implemented by governments to reduce the spread of Covid-19. These include vaccine passports, essential wearing of face coverings, lateral flow testing, evening curfews and even further lockdowns. it is important, moving forward, that the disruption Covid-19 placed on cancer services is not repeated and patients feel they can present to their GP with potential cancer symptoms, investigations are readily available, and treatment is ongoing in a safe and efficient manner.

### Public health campaigns

As we have discussed the number of patients presenting to primary care significantly reduced during the initial lockdown. Despite lockdown easing and individuals being encouraged to attend their GP the number of urgent suspicion of cancer referrals for lung cancer continues to be below the expected levels.

It is vital, therefore, that the general public knows the symptoms of lung cancer, how to seek help for these symptoms, and to be reminded that not all symptoms are attributable to Covid-19. Also, that the public is aware that GP practices are open and willing to see patients. Multiple campaigns have been implemented to encourage the public to seek healthcare when needed throughout the pandemic. This included the ‘Help Us Help You’ campaign by Public Health England^49^ and the ‘Detect Cancer Early—Don’t let lung cancer make itself at home’ campaign from the Scottish Government^50^. Both these campaigns had a focus on lung cancer, encouraging individuals to present to their GP if they had a new persistent cough for more than 3 weeks or unusual breathlessness.

Previous campaigns such as the ‘Be Clear on Cancer’ campaign in England resulted in an increase of 32% in 2-week-wait referrals for suspected lung cancer during the campaign^51^. Similar campaigns have also demonstrated a lung cancer detection shift to earlier more treatable disease^52^.

Utilising such campaigns is more problematic during a pandemic when health services are already stretched and at the time of writing staff absences due to Covid-19 are at an all-time high. Campaigns inevitably result in some patients presenting with minor symptoms; targeting hard to reach individuals with significant symptoms is always challenging. While the evidence is suggestive of increased consultations and a modest improvement in detection of lung cancers at an earlier stage^53^ there is limited evidence for impact on survival; accordingly, it is important that campaigns are implemented with caution, targeting the highest risk individuals^54^.

### Primary care response to respiratory symptoms during and after the Covid-19 pandemic

Early recognition of lung cancer symptoms is challenging in primary care with patients often presenting several times before an onward referral^55^. A full-time equivalent GP will only see one or two new lung cancer cases per year versus hundreds of patients with similar symptoms due to other more often benign conditions—so there can be significant difficulties in early recognition^56,57^.

These difficulties were inevitably exacerbated by the Covid-19 pandemic; in recognition of this, guidance was issued to UK GPs on distinguishing between patients with Covid-19, and those with other respiratory conditions^58,59^.

Effective safety netting has gained further salience with the pandemic, especially when consultations have been conducted remotely^60^. It will also be important going forward that the right balance between telephone/e-consulting, video consulting and face to face consultations is achieved. To do this investment in safe and effective PPE is vital to allow face to face consultations to be carried out safely^61^. Increased funding and recruitment to primary care is essential to ensure the increasing demand can be met and the right type of consultation is able to be offered to individuals^62,63^. it is important that opportunities for early, symptom-based diagnosis in primary care are maximised—ultimately the imperative is to diagnose lung cancer at an early stage. However, these strategies will not, in themselves, lead to substantial stage-shifts, as many lung cancers only produce symptoms at a late stage. This underlines the importance of primary prevention, and research into biomarkers and screening.

### Lung cancer screening

Screening looks to be one of the most promising interventions in detecting lung cancer at an earlier stage where more treatment options are available. The use of low-dose CT as a screening tool targeted at individuals who are considered high risk has significant promise and the most recent updated meta-analysis of randomised control trials demonstrated conclusively a reduction in mortality^64^. This included large scale international screening trials such as The US National lung screening trial (NLST) which demonstrated a 20% reduction in lung cancer mortality and the NELSON trial which demonstrated an even greater reduction in mortality of 24% in men and 33% in women who underwent screening in comparison to controls.

It is important that strategies on how best to implement screening here in the UK are reviewed and focus paid on how to target hard to reach individuals less likely to engage. Individuals from more deprived backgrounds are less likely to participate in cancer screening generally^65–67^ and an analysis of the UK lung cancer screening trial (UKLS) identified that individuals who are active smokers, female and those from lower socioeconomic group were less willing to participate^68^. The most common barriers described included travel, own co-morbidities and caring responsibilities, alongside emotional barriers such as fear and avoidance. Strategies to attempt to overcome these barriers may include community engagement projects, working with primary care practitioners to promote opportunistic discussions with hard-to-reach individuals, mobile screening sites and public health media campaigns.

The pandemic led to many screening programmes around the world being put on hold, and imaging capacity (specifically low-dose CT scanning) is still limited in many regions. However, screening is one potential strategy to deal with the Covid-related ‘backlog’ of undiagnosed lung cancer in many regions of the world, and there is a growing recognition that the harmful effects of Covid-19 are augmented if they lead to reduced recognition and treatment of other illnesses.

There is no national lung screening programme at present in the UK; while a number of pilots are underway, lung cancer screening is still some way off in the UK, although it is under consideration by its National Screening Committee. There are significant hurdles—including imaging capacity, workforce considerations and reluctance, particularly amongst more deprived groups, to accept offers of screening^69^. it is also important to take a cautious approach to implementation of lung cancer screening—its benefits are confined to higher-risk groups and, as with any form of cancer screening, there is potential for over-diagnosis, particularly if recruitment to screening is not sufficiently targeted^70^. Further, it is important to acknowledge the ongoing debate over lung cancer screening. While it is made its way into some national recommendations^71,72^, concerns remain over cost-effectiveness and the potential for over-diagnosis^73^. These are important questions which are the subject of on-going pilots in Europe^74^.

## Conclusions

At the time of writing the worst of the threat from the omicron variant has hopefully passed. But new variants continue to carry the risk of derailing the precarious recovery many lung cancer services around the world were beginning to make. it is important, in primary care, that we do not repeat the mistakes of the early stages of the pandemic; we must take all the steps we can to ensure that patients with symptoms of cancer are seen and referred in a timely way. We need to avail ourselves of new strategies designed to expedite diagnosis, such as the rapid diagnosis centres developing around the UK^75^. It is important that we join public health efforts to ensure that the right balance is achieved between Covid-related precautionary measures, and presentation with symptoms which may be the first indication of lung cancer. Otherwise, we risk a far more prolonged ‘knock-on’ effect from the pandemic, with later-stage presentation and reduced survival.

## References

[1] Cancer Research UK. *Lung Cancer Statistics*. https://www.cancerresearchuk.org/health-professional/cancer-statistics/statistics-by-cancer-type/lung-cancer#heading-One (2021).

[2] Office of National Statistics. *Cancer Survival by Stage at Diagnosis for England* (2019).

[3] Dubey AK, Gupta U, Jain S (2016). Epidemiology of lung cancer and approaches for its prediction: a systematic review and analysis. Chin. J. Cancer.

[4] De Angelis R (2014). Cancer survival in Europe 1999–2007 by country and age: results of EUROCARE-5—a population-based study. Lancet Oncol..

[5] Francisci S (2015). Survival patterns in lung and pleural cancer in Europe 1999–2007: results from the EUROCARE-5 study. Eur. J. Cancer.

[6] Allemani C (2018). Global surveillance of trends in cancer survival 2000-14 (CONCORD-3): analysis of individual records for 37 513 025 patients diagnosed with one of 18 cancers from 322 population-based registries in 71 countries. Lancet.

[7] Walters S (2013). Lung cancer survival and stage at diagnosis in Australia, Canada, Denmark, Norway, Sweden and the UK: a population-based study, 2004–2007. Thorax.

[8] Coalition, U. K. L. C. COVID-19 Matters—a review of the impact of COVID-19 on the lung cancer pathway and opportunities for innovation emerging from the health system response to the pandemic. https://www.uklcc.org.uk/sites/default/files/2021-06/UKLCC-COVID-19-Matters-Report.pdf (2020).

[9] Rodak O, Peris-Díaz MD, Olbromski M, Podhorska-Okołów M, Dzięgiel P (2021). Current landscape of non-small cell lung cancer: epidemiology, histological classification, targeted therapies, and immunotherapy. Cancers.

[10] World Health Organisation. *Covid-19 Significantly Impacts Health Services for Non-communicable Diseases* (2020).

[11] Round T (2021). COVID-19 and the multidisciplinary care of patients with lung cancer: an evidence-based review and commentary. Br. J. Cancer.

[12] Shankar A (2020). Lung cancer management challenges amidst COVID-19 pandemic: hope lives here. Lung Cancer Manag..

[13] Sha Z (2020). The impact of the COVID-19 pandemic on lung cancer patients. Ann. Palliat. Med..

[14] Haldane V (2020). National primary care responses to COVID-19: a rapid review of the literature. BMJ Open.

[15] Due TD, Thorsen T, Andersen JH (2021). Use of alternative consultation forms in Danish general practice in the initial phase of the COVID-19 pandemic—a qualitative study. BMC Fam. Pr..

[16] NHS England. *Advice on How to Establish a Remote ‘Total Triage’ Model in General Practice Using Online Consultations* (2020).

[17] Murchie P (2020). Cancer diagnosis in Scottish primary care: results from the National Cancer Diagnosis Audit. Eur. J. Cancer Care.

[18] Swann R (2018). Diagnosing cancer in primary care: results from the National Cancer Diagnosis Audit. Br. J. Gen. Pract..

[19] Menon U (2019). Time intervals and routes to diagnosis for lung cancer in 10 jurisdictions: cross-sectional study findings from the International Cancer Benchmarking Partnership (ICBP). BMJ Open.

[20] Archer, S. et al. Impact of the COVID-19 pandemic on cancer assessment in primary care: a qualitative study of GP views. *BJGP Open* **5**, BJGPO.2021.0056 (2021). 10.3399/BJGPO.2021.0056 PMC8450883 34006530

[21] Gray DP, Freeman G, Johns C, Roland M (2020). Covid 19: a fork in the road for general practice. BMJ.

[22] Murphy M (2021). Implementation of remote consulting in UK primary care following the COVID-19 pandemic: a mixed-methods longitudinal study. Br. J. Gen. Pr..

[23] Scott LJ (2021). Changes in presentations with features potentially indicating cancer in primary care during the COVID-19 pandemic: a retrospective cohort study. BMJ Open.

[24] Quinn-Scoggins HD (2021). Cancer symptom experience and help-seeking behaviour during the COVID-19 pandemic in the UK: a cross-sectional population survey. BMJ Open.

[25] Healthwatch. *GP Access During COVID-19—A Review of Our Evidence: April 2019–December 2020*. https://www.healthwatch.co.uk/sites/healthwatch.co.uk/files/20210215%20GP%20access%20during%20COVID19%20report%20final_0.pdf (2021).

[26] Gray DP, Sidaway-Lee K, Harding A, Evans P (2020). Reduction in face-to-face GP consultations. Br. J. Gen. Pract..

[27] Homeniuk R, Collins C (2021). How COVID-19 has affected general practice consultations and income: general practitioner cross-sectional population survey evidence from Ireland. BMJ Open.

[28] Friedemann Smith C (2020). GPs’ use of gut feelings when assessing cancer risk in primary care: a qualitative study. Br. J. Gen. Pract..

[29] Smith CF, Drew S, Ziebland S, Nicholson BD (2020). Understanding the role of GPs’ gut feelings in diagnosing cancer in primary care: a systematic review and meta-analysis of existing evidence. Br. J. Gen. Pract..

[30] Vasilakis C, Forte P (2021). Setting up a rapid diagnostic clinic for patients with vague symptoms of cancer: a mixed method process evaluation study. BMC Health Serv. Res..

[31] Dolly SO (2021). The effectiveness of the Guy’s Rapid Diagnostic Clinic (RDC) in detecting cancer and serious conditions in vague symptom patients. Br. J. Cancer.

[32] Norman, C., Wildman, J. M. & Sowden, S. COVID-19 at the deep end: a qualitative interview study of primary care staff working in the most deprived areas of England during the COVID-19 pandemic. *Int. J. Environ. Res. Public Health* **18**, 10.3390/ijerph18168689 (2021). 10.3390/ijerph18168689 PMC8393368 34444437

[33] Powell HA (2019). Socioeconomic deprivation and inequalities in lung cancer: time to delve deeper?. Thorax.

[34] Hovanec J (2018). Lung cancer and socioeconomic status in a pooled analysis of case-control studies. PLoS ONE.

[35] Redondo-Sánchez D (2022). Socio-economic inequalities in lung cancer outcomes: an overview of systematic reviews. Cancers.

[36] National Institute for Health and Care Excellence (NICE). *Diagnosis and Staging of Lung Cancer*. https://pathways.nice.org.uk/pathways/lung-cancer#path=view%3A/pathways/lung-cancer/diagnosis-and-staging-of-lung-cancer.xml&content=view-node%3Anodes-symptoms-and-signs-indicating-urgent-chest-x-ray-and-urgent-and-immediate-referral (2019).

[37] Scottish Cancer Referral Guidelines. *Lung Cancer*. http://www.cancerreferral.scot.nhs.uk/lung-cancer/ (2019).

[38] Crawford SM, Evans C, Edwards H, Zoltowski A (2021). Requests from primary care for chest X-ray and CA125 measurements during the COVID-19 emergency: An observational study. Clin. Med..

[39] Cancer Research UK. *One Year on: How has Covid 19 Affected Cancer Services?*. https://news.cancerresearchuk.org/2021/05/14/one-year-on-how-has-covid-19-affected-cancer-services/ (2021).

[40] Cancer Research UK. *Health Professional COVID-19 and Cancer Hub.* https://www.cancerresearchuk.org/health-professional/diagnosis/hp-covid-19-and-cancer-hub#HP_COVID-191 (2021).

[41] Mahase E (2020). Covid-19: Urgent cancer referrals fall by 60%, showing “brutal” impact of pandemic. BMJ.

[42] Davies, J. What Impact has COVID-19 had on cancer services? Nuffield trust. https://www.nuffieldtrust.org.uk/news-item/what-impact-has-covid-19-had-on-cancer-services (2021).

[43] Skovlund CW, Friis S, Dehlendorff C, Nilbert MC, Mørch LS (2021). Hidden morbidities: drop in cancer diagnoses during the COVID-19 pandemic in Denmark. Acta Oncologica.

[44] Dinmohamed AG (2020). Fewer cancer diagnoses during the COVID-19 epidemic in the Netherlands. Lancet Oncol..

[45] Maluchnik M, Podwójcic K, Więckowska B (2021). Decreasing access to cancer diagnosis and treatment during the COVID-19 pandemic in Poland. Acta Oncologica.

[46] Public Health Scotland. *Cancer Staging Data Using 2018–2020 DCE Data—The Impact of COVID-19*. https://www.publichealthscotland.scot/publications/cancer-staging-data-using-2018-to-2020-dce-data-the-impact-of-covid-19/cancer-staging-data-using-2018-to-2020-dce-data-the-impact-of-covid-19/ (2021).

[47] Mynard, N. et al. Lung cancer stage shift as a result of COVID-19 lockdowns in New York City, a brief report. *Clin. Lung Cancer*, 10.1016/j.cllc.2021.08.010 (2021). 10.1016/j.cllc.2021.08.010 PMC8403338 34580031

[48] Baldwin, D. R. *COVID-19 and Lung Cancer: A Call for Immediate Action*. International Association for the Study of Lung Cancer (IASLC). https://www.iaslc.org/iaslc-news/ilcn/covid-19-and-lung-cancer-call-immediate-action (2021).

[49] Public Health England. *Help Us Help You—Lung Cancer Symptoms*. https://campaignresources.phe.gov.uk/resources/campaigns/120-help-us-help-you---lungcancer-symptoms- (2020).

[50] NHS Greater Glasgow and Clyde. *Don’t Let Lung Cancer Make Itself Home*. https://www.nhsggc.org.uk/about-us/media-centre/news/2021/06/don-t-let-lung-cancer-make-itself-at-home/# (2021).

[51] Ironmonger L (2015). An evaluation of the impact of large-scale interventions to raise public awareness of a lung cancer symptom. Br. J. Cancer.

[52] Kennedy MPT (2018). Lung cancer stage-shift following a symptom awareness campaign. Thorax.

[53] Ball S (2022). An evaluation of a national mass media campaign to raise public awareness of possible lung cancer symptoms in England in 2016 and 2017. Br. J. Cancer.

[54] Lai J (2021). Reviewing the impact of 11 national Be Clear on Cancer public awareness campaigns, England, 2012 to 2016: a synthesis of published evaluation results. Int. J. Cancer.

[55] Lyratzopoulos G, Neal RD, Barbiere JM, Rubin GP, Abel GA (2012). Variation in number of general practitioner consultations before hospital referral for cancer: findings from the 2010 National Cancer Patient Experience Survey in England. Lancet Oncol..

[56] Walter FM (2015). Symptoms and other factors associated with time to diagnosis and stage of lung cancer: a prospective cohort study. Br. J. Cancer.

[57] Weller DP, Peake MD, Field JK (2019). Presentation of lung cancer in primary care. NPJ Prim. Care Respir. Med..

[58] Scottish Government. *COVID-19: The Management of Urgent Suspicion of Lung Cancer Referrals*. https://www.gov.scot/publications/coronavirus-covid-19-management-of-urgent-suspicion-of-lung-cancer-referrals/ (2020).

[59] British Thoracic Society. *Differentiation of the Cs in Lung Cancer: Cancer vs. COVID 2020* (2020).

[60] Greenhalgh T, Koh GCH, Car J (2020). Covid-19: a remote assessment in primary care. BMJ.

[61] Torjesen I (2021). Covid-19: PPE guidance is upgraded as evidence of airborne transmission grows. BMJ.

[62] Mahase E (2021). GPs are being blamed for government failures in primary care, say doctors. BMJ.

[63] Hodes, S. et al. If general practice fails, the NHS fails. *BMJ Opinion* (2021).

[64] Field, J. K. et al. Lung cancer mortality reduction by LDCT screening: UKLS randomised trial results and international meta-analysis. *The Lancet Regional Health – Europe* **10**, 10.1016/j.lanepe.2021.100179 (2021). 10.1016/j.lanepe.2021.100179 PMC8589726 34806061

[65] Hestbech MS, Siersma V, Dirksen A, Pedersen JH, Brodersen J (2011). Participation bias in a randomised trial of screening for lung cancer. Lung Cancer.

[66] Pornet C, Dejardin O, Morlais F, Bouvier V, Launoy G (2010). Socioeconomic determinants for compliance to colorectal cancer screening. A multilevel analysis. J. Epidemiol. Community Health.

[67] Lal N, Singh HK, Majeed A, Pawa N (2021). The impact of socioeconomic deprivation on the uptake of colorectal cancer screening in London. J. Med. Screen.

[68] Ali N (2015). Barriers to uptake among high-risk individuals declining participation in lung cancer screening: a mixed methods analysis of the UK Lung Cancer Screening (UKLS) trial. BMJ Open.

[69] van der Aalst CM, ten Haaf K, de Koning HJ, 4-IN-THE-LUNG-RUN Consortium (2020). Implementation of lung cancer screening: what are the main issues?. Transl. Lung Cancer Res..

[70] Callister MEJ, Sasieni P, Robbins HA (2021). Overdiagnosis in lung cancer screening. Lancet Respiratory Med..

[71] Krist AH (2021). Screening for lung cancer: US Preventive Services Task Force recommendation statement. Jama.

[72] Kauczor H-U (2020). ESR/ERS statement paper on lung cancer screening. Eur. Radiol..

[73] Brodersen J (2020). Overdiagnosis of lung cancer with low-dose computed tomography screening: meta-analysis of the randomised clinical trials. Breathe.

[74] van Meerbeeck JP, Franck C (2021). Lung cancer screening in Europe: where are we in 2021?. Transl. Lung Cancer Res..

[75] Sewell B (2020). Rapid cancer diagnosis for patients with vague symptoms: a cost-effectiveness study. Br. J. Gen. Pr..

